# Examining the approach to medical remediation programmes—an observational study

**DOI:** 10.1007/s11845-024-03654-0

**Published:** 2024-03-13

**Authors:** Sean Maher, Stephanie Ryan, Conor O’Brien, Daniel Fraughen, Muirne Spooner, Noel G. McElvaney

**Affiliations:** 1 University of Medical and Health Sciences, Royal College of Surgeons, Dublin, Ireland; 2 Beaumont Hospital, Dublin, Ireland

**Keywords:** Health, Ireland, Medical education, Remediation programmes

## Abstract

**Background:**

Remediation of underperforming students is recognised as an important tool in medical education; however, there is no universally agreed approach.

**Aims:**

This study aimed to evaluate the effectiveness of a remediation program for final year medical students who failed their first long case assessment (LCA1) and to compare their academic performance with their peers who passed their first long case assessment.

**Methods:**

The study consisted of two phases. Phase 1 analysed the demographics and academic performance data for the 9% of the class in the remediation group. Phase 2 focused on collecting similar data for the remaining 91% of students in the non-remediation group. Statistical analyses including the Wilcoxon rank sum test and Pearson correlation coefficients were used to compare the groups.

**Results:**

Phase 1 showed 88% of students who participated in remediation successfully passed the second long case assessment (LCA2); however, 25% of this cohort ultimately failed the academic year due to poor results in other assessments. Phase 2 results revealed that non-remediation group students scored significantly higher in LCA2 (59.71% vs 52.07%, *p* < 0.001) compared to their remediation counterparts, despite 19% of them failing this assessment. Non-remediation group students consistently outperformed their remediation group counterparts in formative and summative assessments. Overall, 6.25% of the entire class failed the academic year.

**Conclusion:**

This study demonstrates the need to focus on overall academic performance to identify struggling students rather than one high stakes exam. Most of the students in the remediation programme ultimately passed LCA2.

## Introduction

Medical education is a demanding and challenging process that requires students to acquire a significant body of knowledge and practical skills [1]. Academic success is complex and is linked to both individual and external learning factors such as system resources and student motivation [2]. Medical education is unique in that if a doctor is underperforming, patients may be at risk [3], and thus, remediation is a key tool in ensuring graduates meet an exacting required standard [4].

Despite its importance in the regulation of knowledge and ensuring minimum safe standards across medical school graduates, there is no standardised approach to remediation [5]. Steinert outlined a structured approach to the “problem-learner” where she defined effective interventions such as enhanced teaching and learning opportunities, and peer or mentor support [6]. However, she concluded that teachers and system factors must also be considered in the learning process and not just the student alone. A literature review by Hauer (2009) found a paucity of evidence to guide the best practice for remediation in medical education at undergraduate, graduate and continuing medical education levels [7]. Ten years later, a further literature review by Brennan et al. [8] looked at 19 studies targeting remediating professionalism in doctors, medical students, residents, and mixed populations. However, this review showed no ideal approach or best practice on remediation interventions and suggested that further research was needed [8].

The long case assessment (LCA) is designed to assess the students’ ability to apply their knowledge and clinical exam skills to real-life scenarios in the latter years of their medical training [9]. In our institution, each final year medical student will complete two LCAs, one in the first semester (worth 40%) and another in the second semester (worth 60%). The LCA involves the student taking a history from a patient in front of two examiners. The student then presents their findings and gives a differential diagnosis. They are then asked to examine a body system relevant to the patient’s history and finally the student is asked to discuss patient investigations and management. Overall, the LCAs account for 35% of the medical and surgical grade. The LCA is also a “must pass” component overall. Presently, only students who fail their first LCA are enrolled in the remediation programme. In this, the students are offered additional bedside and classroom-based tutorials, with a specific focus on improving performance in the second LCA.

This study set out to assess the effectiveness of the remediation programme in its current form and to establish if enrolment led to improved second LCA results. Secondly, we aimed to establish if other variables such as age, sex, and topic examined, or previous academic performance influenced LCA outcome. Finally, the study sought to examine whether the inclusion criteria for our remediation programme were appropriate and whether we were capturing all the students who most needed additional teaching.

In the absence of clear guidelines and an agreed approach to remediation [5, 10] we sought to examine our own practices to identify gaps in teaching models and, by evaluating the effectiveness of our current remediation programme, determine how best to intervene to improve student experience and success.

## Methods

### Phase 1: remediation group recruitment and analysis

In phase 1, all final year (SC2) medical students who failed their first LCA in December were assigned to a remediation programme. In the remediation programme, students were offered both extra bedside and classroom-based tutorials which were directed towards improving second LCA performance. We collected data on these students’ LCA topic, clinical site, exam score (numerical grade) and exam result (pass/fail). We documented the number of remediation tutorials offered to each student, the percentage of these tutorials that each student attended, and the hospital where the tutorials took place (Beaumont Hospital, Connolly Hospital and Our Lady of Lourdes, Drogheda). In addition, we also recorded baseline demographics such as student age and sex, as well as previous attendance at small group tutorials (SGTs) and academic performance to date. Academic performance included any assessment completed prior to their first LCA such as, their end-of-year result in their penultimate year of study (SC1), and scores from final year (SC2) summative written and skills-based examinations. Once the remediation programme was complete, we also recorded results from these students’ second LCA in April.

### Phase 2: non-remediation group recruitment and analysis

In phase 2, we examined the demographics and academic performance of all students in final year (SC2). This group was designated the “non-remediation group”, and the same data were collected for this group, apart from remediation tutorials they attended.

### Statistical analysis

The characteristics of the groups were described with (%) or % ($\pm {\text{SD}})$. Differences between two groups means were tested using a Wilcoxon rank sum test. Differences in the proportions were tested for using chi-squared test or Fischer exact test. We assessed for correlations using the Pearson correlation coefficient.

## Results

### Effectiveness of the remediation

**Fig. 1:**
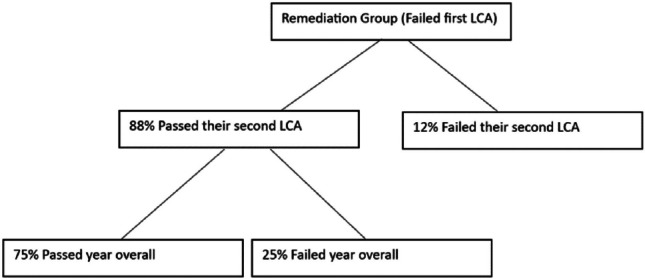
88% of students who failed their first LCA passed the second LCA

Six extra tutorials were offered over a seven-week period, and the attendance rate was 54% across the three clinical sites. The majority of the students who went through the remediation program subsequently went on to pass their second LCA (88%) (Fig. 1).

Of those who failed the final year exam overall, 50% had been in the remediation program. Despite 88% of students in the remediation group obtaining a passing grade in their second LCA, 25% of this group did not reach a passing grade overall. This was due to failure in other components of the overall exam. For example, those who failed the second LCA had a significantly lower score in their surgical MCQ examination (54.62 vs 44.28, *p* = 0.046).

**Fig. 2:**
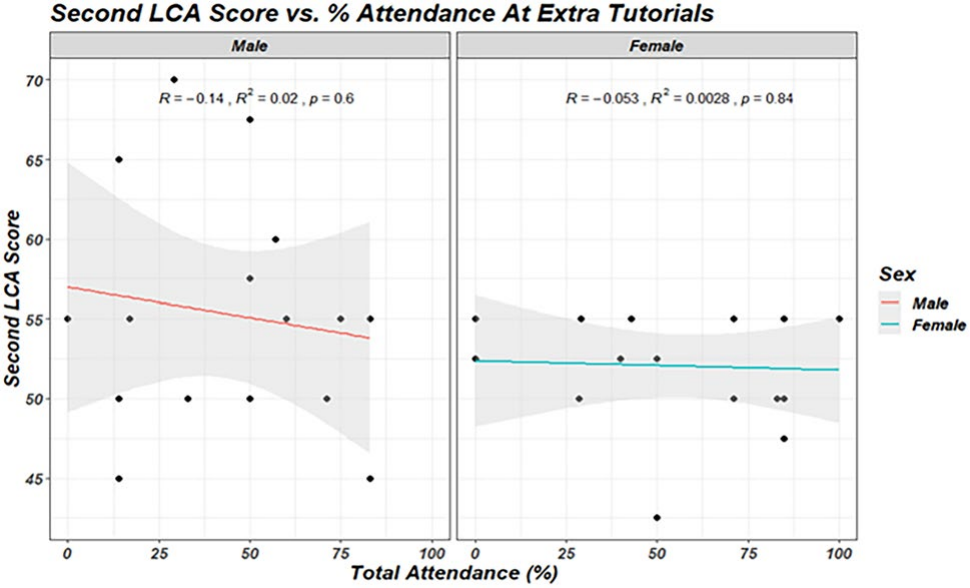
Correlation between second LCA score and attendance (%) at extra tutorials in male and female students

Neither the total number of extra tutorials offered to students, nor their percentage attendance at these tutorials had a statistically significant effect on their second LCA results (*p* = 0.624). Percent attendance at tutorials was not found to statistically impact second LCA scores (Fig. 2).

Factors such as sex and examination site were not found to have a statistically significant effect on second LCA result or score in the remediation cohort.

### Picking the right people for remediation: comparison with the overall class

In phase 2, we combined the non-remediation students with the remediation students to assess whether the results obtained in phase 1 were applicable to the entire year group. We found that students in the non-remediation group scored significantly higher in second LCA (59.71% vs 52.07%, *p* < 0.001). However, 83% of students who failed LCA2 were in the non-remediation group.

The non-remediation group was found to have consistently performed better in assessments throughout the academic year when compared to the remediation group (Table 1).

**Table 1:** Raw assessment scores in remediation vs non-remediation groups across different assessment modalities. Data by long case assessment 2 results (pass [≥ 50]/fail [< 50])

**Characteristic**	**Whole class**	**Long case 2 result**		***p*-value**
		**Pass (93%)**	**Fail (7%)**	
**Medicine MLC score**	56.95 (7.48)	57.32 (7.46)	53.53 (7.02)	0.012^a^
**Medicine TOSBA 1 score**	58.18 (6.88)	58.40 (6.89)	55.00 (6.03)	0.007^a^
**Medicine TOSBA 2 score**	61.34 (6.26)	61.60 (6.22)	57.61 (5.81)	0.003^a^
**Surgical TOSBA 1 score**	59.42 (8.51)	59.79 (8.42)	54.13 (8.21)	< 0.001^a^
**Surgical TOSBA 2 score**	60.65 (8.02)	61.06 (7.91)	54.78 (7.30)	< 0.001^a^
**Medicine MCQ score**	63.73 (15.31)	64.87 (14.75)	47.40 (14.08)	< 0.001^a^
**Surgical MCQ score**	63.90 (10.58)	64.72 (10.11)	52.14 (10.41)	< 0.001^a^
**OSCE score**	69.23 (9.31)	69.85 (9.11)	60.39 (7.71)	< 0.001^a^
**Medicine case presentation 1 score**	61.38 (8.09)	61.82 (8.10)	55.00 (4.52)	< 0.001^a^
**Medicine case presentation 2 score**	62.34 (8.89)	62.90 (8.76)	54.35 (6.79)	< 0.001^a^
**Surgical case presentation 1 score**	63.75 (8.03)	64.03 (8.08)	59.78 (6.12)	0.005^a^
**Surgical case presentation 2 score**	65.28 (7.48)	65.91 (6.49)	56.30 (13.16)	< 0.001^a^
**SUBI score**	84.42 (16.54)	85.20 (15.97)	73.46 (20.70)	< 0.001^a^

^a^Wilxocon rank sum test

Females performed significantly better in both first LCA (60.16% vs 57.91%, *p* = 0.009) and second LCA (61.21% vs 59.21%, *p* = 0.039), as well as other forms of in-person assessment, such as Surgical TOSBAs 1 and 2, Medicine Case Presentation 2, and Surgical Case Presentations 1 and 2.

Female sex had no statistically significant impact on other forms of in-person and written assessment, including the Medicine Mock Long Case, Medicine Case Presentation 1, Medicine TOSBAs 1 and 2, Medicine MCQ, Surgery MCQ, or SUBI Assessment. The reasons for these gender differences are unclear.

### Students who failed the second LCA but passed the first LCA

Looking specifically at the cohort of students who passed the first LCA and subsequently failed the second LCA; they were significantly weaker compared to the overall class across practically all summative assessments throughout the year (Table 2).

**Table 2:** Summative assessment scores of students who passed first long case and failed second long case compared to rest of the class

**Characteristic**	**Whole class**	**Failed LCA2 but passed LCA1**		***p*-value**
		**Yes = 5%**	**No = 95%**	
**Medicine MLC score**	57.02 (7.53)	57.57 (7.70)	57.32 (7.46)	0.035^a^
**Medicine TOSBA 1 score**	58.23 (6.89)	55.26 (6.34)	58.40 (6.89)	0.027^a^
**Medicine TOSBA 2 score**	61.35 (6.28)	57.11 (6.08)	61.60 (6.22)	0.002^a^
**Surgical TOSBA 1 score**	59.48 (8.51)	54.21 (8.70)	59.79 (8.42)	0.002^a^
**Surgical TOSBA 2 score**	60.79 (7.95)	56.05 (7.37)	61.06 (7.91)	0.002^a^
**Medicine MCQ score**	64.01 (15.10)	49.07 (13.55)	64.87 (14.75)	< 0.001^a^
**Surgical MCQ score**	64.12 (10.40)	53.80 (10.08)	64.72 (10.11)	< 0.001^a^
**OSCE score**	69.23 (9.34)	58.55 (6.66)	69.85 (9.11)	< 0.001^a^
**Medicine case presentation 1 score**	61.45 (8.10)	55.00 (4.71)	61.82 (8.10)	< 0.001^a^
**Surgical case presentation 1 score**	63.81 (8.03)	60.00 (6.01)	64.03 (8.08)	0.012^a^
**Surgical case presentation 2 score**	65.36 (7.46)	55.79 (14.27)	65.91 (6.49)	< 0.001^a^

^a^Wilxocon rank sum test

### Overall failing grade

6.25% of the class failed the year overall. Failing students were also more likely to have failed their penultimate year (SC1). A statistically significant proportion had failed their medical mock long case compared with the rest of the class, whilst Surgical TOSBA results were significantly lower between the failing and passing groups. These characteristics are summarised in Table 3 below.

**Table 3:** Academic characteristics of students who failed the year overall

Failed overall (6% of whole class)		
	**% total**	**p-value**
**SC1 result**		0.011^a^
*Pass*	91%	
*Fail*	9%	
**Medicine MLC result**		< 0.001^a^
*Pass*	60%	
*Fail*	40%	
**Surgical TOSBA 1 result**		0.005^a^
*Pass*	73%	
*Fail*	27%	
**Surgical TOSBA 2 result**		< 0.001^a^
*Pass*	77%	
*Fail*	23%	
**Medicine MCQ result**		< 0.001^b^
*Pass*	5%	
*Fail*	95%	
**Surgical MCQ result**		< 0.001^b^
*Pass*	14%	
*Fail*	86%	

^a^Fischer extract test

^b^Pearson’s chi-squared test

## Discussion

Most students enrolled in the remediation program went on to pass the second LCA. However, attendance at an increased number of tutorials did not significantly correlate with improved scores at this LCA. Student success relies on a complex range of factors [11] and might not be due to teaching hours alone. However, another possible explanation might be that the stronger students may have been less likely to attend the tutorials, whilst students who struggled more could potentially have felt a greater impetus to attend more teaching sessions. Further research is needed to examine the motives for attendance and non-attendance at remediation teaching to establish the impact on academic achievement.

The students within the remediation group performed poorly when compared to their non-remediation classmates across virtually all assessments throughout the course of the academic year and a significant number went on to fail the year overall despite passing the second LCA. Several students who were not part of the remediation group also went on to fail the second LCA, which can potentially lead to an overall fail grade given its relative weighting.

In our cohort, 50% of students who failed overall were not enrolled in the remediation programme, suggesting that the entry criteria for the remediation group may not be effective in identifying the correct students for remediation. Both medical and surgical MCQ results identified practically all those who went on to fail the year overall, with medicine being slightly more discriminating.

Timely identification of struggling students with earlier release of MCQ results could lead to improved provision of targeted academic and pastoral support, giving each student their best opportunity to succeed [12].

Furthermore, students who failed the second LCA after passing the first, also scored lower across other assessments compared to the general class, suggesting warning signs which can be utilised to offer a more effective remediation program. This is supported by the need for frequent and high-quality feedback to guide student performance in their development of the necessary skills and competencies [13].

These data underline the need for a more holistic set of criteria to identify students earlier and offer an opportunity to deliver more targeted interventions. From the data shown in this study, the exam results from as early as the penultimate year may need to be evaluated as students who failed this year were significantly more likely to fail their final year. In the final year, evaluation of the first LCA should be undertaken in conjunction with analysis of summative continuous assessment during the first semester of that year along with the MCQ results also from the first semester. A borderline or fail mark in one or more of these assessments should trigger a remediation review to determine what intervention might work best. It may be that the student may have a knowledge deficit which may not require more patient contact and directed learning may be more applicable and successful in that instance. In students who perform poorly in patient-based scenarios, bedside or patient-related evaluations should be encouraged. In those students with both a knowledge deficit and inability to examine patients and elicit clinical signs it may be that a more intensive “boot camp” is required.

All of this is very labour intensive and the need for additional teaching must be balanced against what is practical to implement from a resource perspective [14]. The more triggers there are for enrolling a student into a remediation program, the larger the cohort of students within the program and subsequent staff required. However, the stakes are high for the student and the general populations they will serve so robust remediation is a necessity not a luxury.

The major strength of this study is that it examines a large and diverse cohort of students across multiple modes of assessment in a medical school in Ireland. To our knowledge, it is the first study to examine such a remediation programme in Ireland and is an important step towards developing a standard approach to remediation. The limitations of this study are that it took place in a single university. The content and format of the remediation tutorial delivered was at the discretion of the tutor/lecturer and was not tailored to specific needs of the student. Furthermore, attendance was not mandatory at the remediation programme and therefore it’s hard to assess its true influence on academic outcomes.

## Conclusion

This study re-demonstrates the lack of consensus guidelines in designing a third level medical remediation programme. Our research shows that we need to focus on more than one aspect of the early exams in determining which students may need remediation. The “early warning signs” demonstrated in this study suggest that whilst some students may have a specific deficit in knowledge and others a deficit in patient encounter-based exams, it is not uncommon for the two to coexist. Frequent examination throughout the year, evaluation of summative and formative assessment results coupled with feedback given at a time where effective remediation can influence results is imperative. This remediation, like the deficits uncovered, may require a more carefully considered student-centric approach in order to succeed.

## Data Availability

Raw data are not publicly available to preserve individuals’ privacy under the European General Data Protection Regulation.

## Abbreviations

TOSBA: Team Objective Structured Bedside Assessment
SUBI: Sub-internship
MCQ: Multiple Choice Question
OSCE: Objective Structured Clinical Examination
MLC: Mock Long Case
